# Differential Proteomic Analysis of Human Saliva using Tandem Mass Tags Quantification for Gastric Cancer Detection

**DOI:** 10.1038/srep22165

**Published:** 2016-02-25

**Authors:** Hua Xiao, Yan Zhang, Yong Kim, Sung Kim, Jae Joon Kim, Kyoung Mee Kim, Janice Yoshizawa, Liu-Yin Fan, Cheng-Xi Cao, David T. W. Wong

**Affiliations:** 1 State Key Laboratory of Microbial Metabolism, Laboratory of Bioseparation and Analytical Biochemistry, School of Life Sciences and Biotechnology, Shanghai Jiao Tong University, Shanghai, 200240, China; 2 School of Pharmacy, Shanghai Jiao Tong University, Shanghai, 200240, China; 3 Dental Research Institute, School of Dentistry, University of California-Los Angeles, Los Angeles, California, 90095, USA; 4 Department of Surgery, Samsung Medical Center, Sungkyunkwan University School of Medicine, Seoul, 030031, Korea; 5 Department of Internal Medicine, Samsung Medical Center, Sungkyunkwan University School of Medicine, Seoul, 030031, Korea; 6 Department of Pathology, Samsung Medical Center, Sungkyunkwan University School of Medicine, Seoul, 030031, Korea

## Abstract

Novel biomarkers and non-invasive diagnostic methods are urgently needed for the screening of gastric cancer to reduce its high mortality. We employed quantitative proteomics approach to develop discriminatory biomarker signatures from human saliva for the detection of gastric cancer. Salivary proteins were analyzed and compared between gastric cancer patients and matched control subjects by using tandem mass tags (TMT) technology. More than 500 proteins were identified with quantification, and 48 of them showed significant difference expression (p < 0.05) between normal controls and gastric cancer patients, including 7 up-regulated proteins and 41 down-regulated proteins. Five proteins were selected for initial verification by ELISA and three were successfully verified, namely cystatin B (CSTB), triosephosphate isomerase (TPI1), and deleted in malignant brain tumors 1 protein (DMBT1). All three proteins could differentiate gastric cancer patients from normal control subjects, dramatically (p < 0.05). The combination of these three biomarkers could reach 85% sensitivity and 80% specificity for the detection of gastric cancer with accuracy of 0.93. This study provides the proof of concept of salivary biomarkers for the non-invasive detection of gastric cancer. It is highly encouraging to turn these biomarkers into an applicable clinical test after large scale validation.

Although the incidence and mortality have fallen dramatically over the past several decades, gastric cancer is still a major public health issue as the fourth most common cancer and the second leading cause of cancer related death worldwide^1^,^2^. Approximately 880,000 people succumb to this malignancy each year on the earth. Radical surgery is the only potential curative method for the localized disease. One of the main reasons for the high mortality is the delay in diagnosis due to the fact that early cancers are typically asymptomatic or causes only nonspecific symptoms^3^. About 90% of gastric cancers are adenocarcinomas. Non-Hodgkin’s lymphomas and leiomyosarcomas make up most of the remaining 10%^4^. *Helicobacter pylori (H. pylori*) infection is the strongest known risk factor for malignancies that arise within the stomach, and epidemiological studies have determined that the attributable risk for gastric cancer conferred by *H. pylori* is about 75%^5^,^6^,^7^,^8^. Usually the 5 year survival rates for gastric cancer are less than 30% in many countries, although it has been significantly improved in some Asian countries by up to 50% because of the early detection programs^9^. The corresponding 5 year survival rates for stage I and II patients are 67%, compared to 31% and 8% in stage III and IV patients, respectively^10^. Therefore, novel biomarkers and non-invasive diagnostic methods are urgently needed for the screening of gastric cancer to reduce the high mortality.

The detection of gastric cancer in the early stages is vitally important in ensuring an excellent prognosis^8^. However, as with other cancers, the challenges in early detection lie in the reality of non-specific symptoms and invasive physical procedures^11^. The symptoms of early stage cancer may be indistinguishable from those of benign dyspepsia, while the presence of alarm symptoms may imply an advanced and often inoperable disease. Dysphagia, weight loss, and a palpable abdominal mass appear to be major independent prognostic factors for gastric cancer. However, gastro-intestinal bleeding, vomiting and duration of symptoms, do not seem to have a relevant prognostic impact on the survival of gastric cancer^3^.

Biomarker discovery for gastric cancer has mainly focused on tissue^12^,^13^, blood^14^,^15^ or gastric juice samples^16^ for the identification of protein^17^, microRNA^18^, long non-coding RNA^19^, and DNA^20^ candidates. For example, serum TIMP-1^15^ has been identified as prognostic biomarkers for gastric cancer. Clinical proteomic study has shown that IPO-38 protein is a promising biomarker both for diagnosis and for predicting prognosis of gastric cancer^17^. Besides, biomarker discovery has been carried out with the treatment of EGFR binding monoclonal antibody in advanced gastric cancer^21^,^22^. However, there are few reliable serum biomarkers for the diagnosis of gastric cancer so far. The available biomarkers of CEA, CA19-9 and CA72-4 are not sufficiently sensitive and specific for the detection of gastric cancer^17^.

Proteomics is a powerful approach for biomedical research. Mapping proteomes from tissues, cells, and organisms is being used to discover new disease biomarkers for clinical and diagnostic applications^23^. Cancer proteomics has been extensively used for the discovery of diagnostic biomarker for gastric cancer^24^,^25^. Different quantitative proteomics have been widely used for biomarker discovery in different types of samples, including surface-enhanced laser desorption/ionization^26^, two-dimensional gel electrophoresis-mass spectrometry^27^,^28^, and isobaric tag for relative and absolute quantitation (iTRAQ)^29^. Proteomics using mass spectrometry with Tandem Mass Tags (TMT) is a reliable technology for quantitative proteome analysis^30^. Each isobaric tagging reagent within a set has the same precursor mass and is composed of an amine-reactive NHS-ester group, a spacer arm and an MS/MS reporter. For each sample, a unique reporter mass results in the MS/MS spectrum (i.e., m/z 126–131 for TMT-6plex Isobaric Label Reagents). These reporter product ions are used to report relative protein expression levels^31^,^32^,^33^.

Endoscopy with biopsy sampling is the gold standard used in gastric cancer diagnosis^34^. However, the invasive and relentless character of this procedure makes it less suitable for fast screening^35^. Human saliva is a biological fluid with enormous diagnostic potentials. Because saliva can be non-invasively collected, it provides an attractive alternative approach for cancer diagnosis. Saliva harbors a wide array of components, especially proteins^36^,^37^, which can be very informative for the detection of oral diseases (e.g. oral cancer^38^ and Sjogren’s Syndrome^39^) and systemic diseases (e.g. breast cancer^40^ and lung cancer^41^). More specifically, saliva protein finger print has been preliminary analyzed for the early diagnosis of gastric cancer^42^. Besides, gram-negative bacterium Helicobacter pylori could secrete enzyme urease and convert urea into carbon dioxide and ammonia. Optoelectronic sensors have been developed to detect clinically relevant levels of carbon dioxide and ammonia in saliva that can potentially be used for early diagnosis of gastric cancer^35^.

In this study, we hypothesized that gastric cancer related proteins exist in human saliva, which could be clinically used to discriminate gastric cancer patients from healthy control subjects. Human saliva samples were collected from gastric cancer patients and matched healthy control subjects. Salivary proteins were analysed and compared between the two groups by using TMT technology for proteomic biomarker identification and quantification. Candidate proteomic biomarkers were selected and further verified by immunoassay. Their utility for the detection of gastric cancer has been evaluated. With the discovery and pre-validation of discriminatory proteomic markers from saliva, gastric cancer will be non-invasively detected with high specificity and sensitivity.

## Results

### The strategy for salivary proteomic biomarker discovery

**Figure 1:**
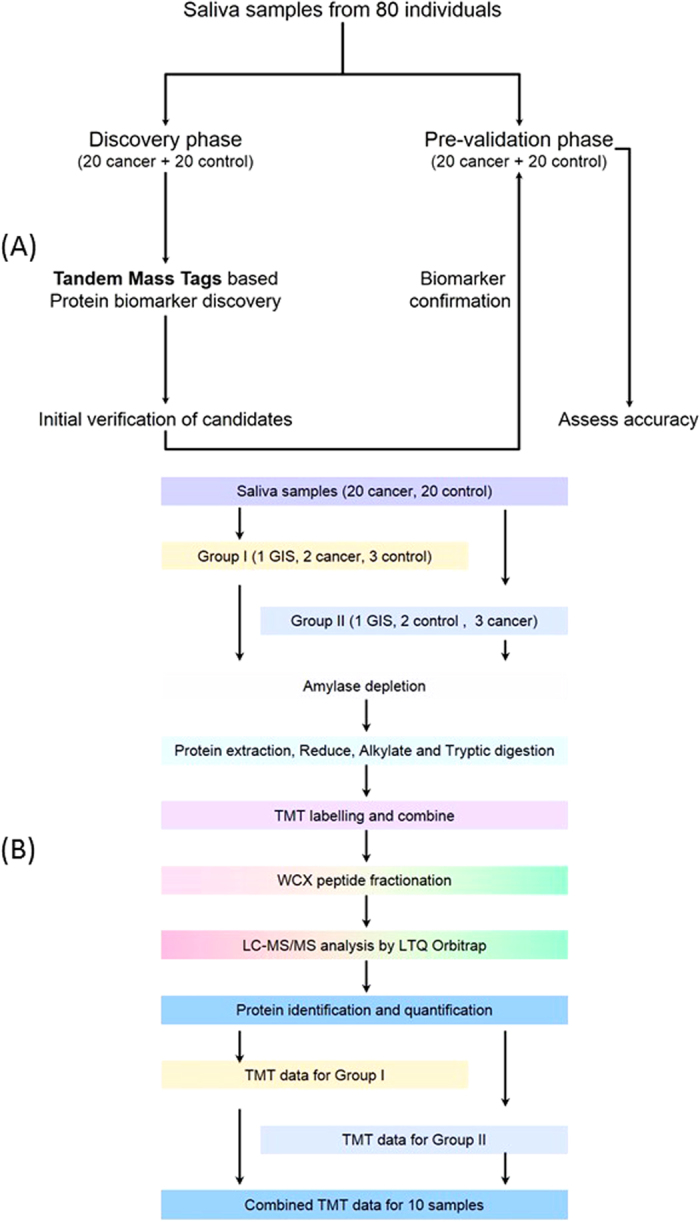
Study design (**A**) and experimental design (**B**) for proteomic biomarker development in human saliva.

The study design is briefly shown in Fig. 1A. The 40 gastric cancer saliva samples were collected from patients who have been diagnosed as gastric cancer by using biopsy at the Samsung Medical Center (Seoul, Korea); most of them were at their early stages, as a result of the National Cancer Screening Program for gastric cancer in Korea^43^. The saliva samples from 40 healthy control subjects were collected as controls by matching their age-, sex-, and ethnicity- with the cancer group. Their smoking and drinking history were matched generally by whether they are current or former smokers and their duration and intensity. Their *H. pylori* infection and intestinal metaplasia status was included. Patient demographics and clinical profiles are present in Table 1.

### Amylase depletion

**Figure 2:**
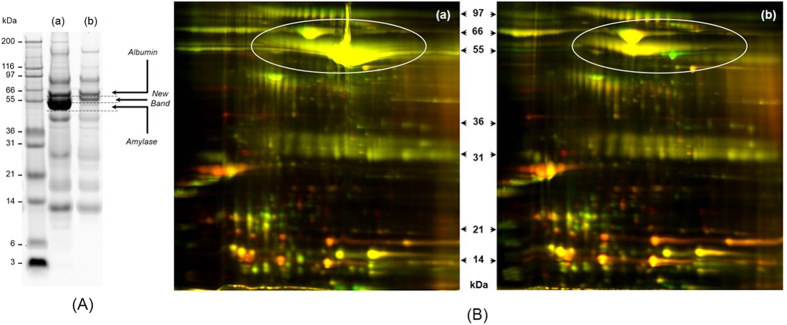
Salivary amylase removal by starch affinity column. (**A**) 1D SDS-PAGE of salivary proteins (**a**) before amylase depletion and (**b**) after amylase depletion; (**B**) 2D-DIGE of salivary proteins before amylase depletion (**a**) and after amylase depletion (**b**); Green: one saliva sample; Red: another saliva sample.

Alpha amylase is the most abundant protein in human saliva, accounts for about 50–60% of the total protein amount, which hurdled the detection and quantification of low abundant proteins. Depletion of these interfering proteins prior to definitive analyses should improve the resolution and sensitivity of salivary proteome analysis^36^. The one-dimensional SDS-polyacrylamide gel electrophoresis (1D SDS-PAGE) of salivary protein profiling before and after amylase depletion is shown in Fig. 2A. Obviously, the dominant band in the saliva without treatment disappeared after flow through the starch column. Figure 2B shows the two-dimensional difference gel electrophoresis (2D-DIGE) of two random saliva samples before and after amylase depletion, the dominant spots labeled in circle in Fig. 2B (a) significantly decreased when compare to that with amylase depletion in Fig. 2B (b).

### Cation exchange peptide fraction and peptides preparation

After amylase depletion, saliva proteins in each sample were reduced, alkylated and then digested by trypsin. According to the assignment in Fig. 1B, each sample in each group was labeled with corresponding tags of TMT-6plex. Combined peptides in group I and group II were fractionated by cation exchange chromatography column into 12 fractions, respectively, as shown in Supplementary Figure S2. All the fractions were dried under vacuum and were rehydrated in mobile phase B for further analysis.

### Identification of differentially expressed proteins in saliva

Each fraction was loaded to liquid chromatography tandem mass spectrometry (LC-MS/MS) for protein identification and quantification. The raw data generated from the 12 fractions in each group was combined for protein database search and analysed in Proteome Discoverer with designed TMT workflow. Briefly, collision-induced dissociation (CID) spectrum was selected from total spectrum and used for protein identification. SEQUEST was interfaced with Proteome Discoverer for protein database search against IPI human database. Higher energy collisional dissociation (HCD) spectrum was extracted from the total spectrum and specifically used for reporter ion quantification. In this study, a global internal standard (GIS) was made and added to each group, specifically the GIS was labelled by TMT m/z at 126.1 in both groups. All the reading of other samples was compared with this GIS, which made the signal of all 10 samples in group I and group II comparable.

The database search results for group I and group II were exported to Microsoft Excel software, including the protein identification and quantification intensity ratios. In total, 519 proteins were identified from all the samples. The quantification data of cancer group and control group was extracted from the corresponding database search results. The distribution of individual proteins in cancer group and control group were systematically compared. 48 proteins showed significant difference (p < 0.05) between cancer group and control group (Table 2). For high throughput biomarker verification and validation, only these gastric cancer related candidates with available ELISA kits were selected for further evaluation.

**Figure 3:**
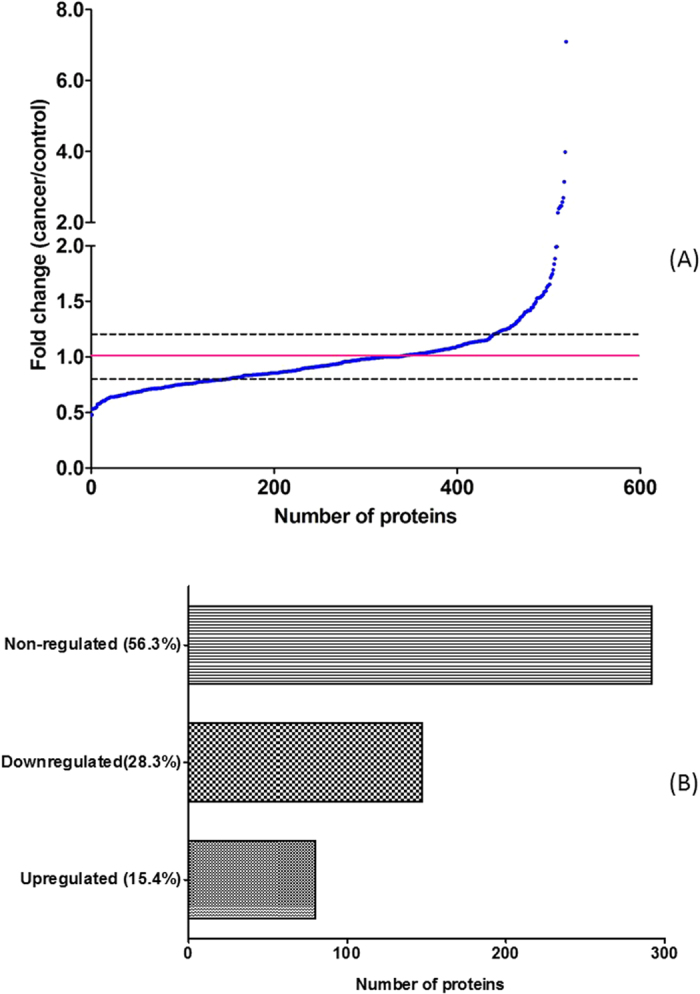
The fold change distribution (**A**) of these quantified proteins between cancer and control group. The regulation of discovered proteins (**B**) in cancer and control groups.

Among these identified 519 proteins, their fold change ranged from 0.48 to 7.9 (Fig. 3A). 292 proteins were quantified with fold change in the range of 0.8 and 1.2. 80 proteins were up-regulated in the cancer group with fold change greater than 1.2. Meanwhile, 147 proteins were down-regulated in the cancer group (Fig. 3B) with fold change less than 0.8.

### TMT based protein identification and quantification

**Figure 4:**
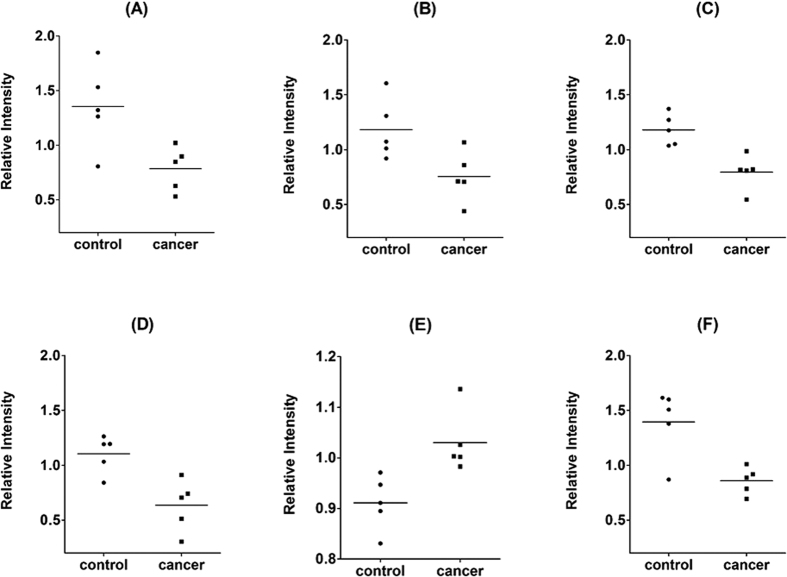
TMT quantification data for six candidate markers (**A**) CSTB, (**B**) TPI1, (**C**) DMBT1, (**D**) CALML3, (**E**) IGH and (**F**) IL1RA.

**Figure 5:**
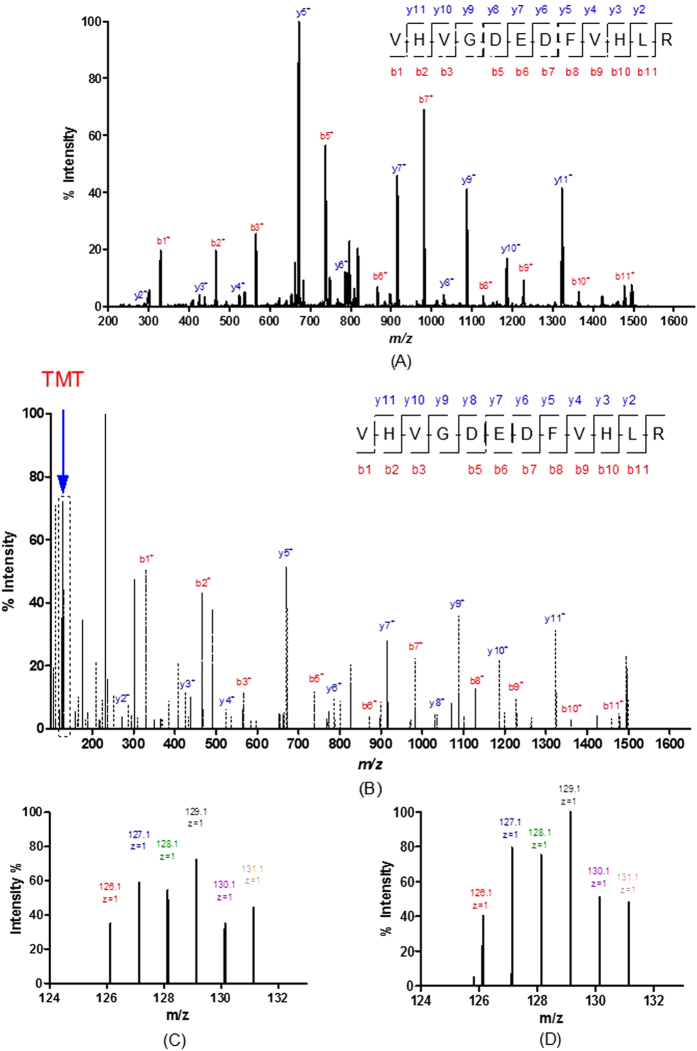
The MS/MS spectra of two peptides for CSTB with TMT labeling in group I: (**A**) CID spectrum for VHVGDEDFVHLR; (**B**) HCD spectrum for VHVGDEDFVHLR; (**C**) TMT reporter spectrum for VHVGDEDFVHLR; m/z = 127.1, 128.1 and 129.1 representing 3 normal samples, m/z = 130.1 and 131.1 representing 2 gastric cancer samples; (**D**) TMT reporter spectrum for peptide VFQSLPHENKPLTLSNYQTNK; m/z = 127.1, 128.1 and 129.1 representing 3 normal samples, m/z = 130.1 and 131.1 representing 2 gastric cancer samples.

The TMT quantification data of 6 candidates biomarker with p < 0.05, including CSTB, TPI1, DMBT1, Calmodulin-like protein 3 (CALML3), Immunoglobulin heavy chain (IGH), Interleukin-1 receptor antagonist (ILIRA), are shown in Fig. 4. The representative MS/MS spectrum of CSTB are shown in Fig. 5. Figure 5A,B are the CID spectrum and HCD spectrum for one peptide of CSTB, respectively. The rectangle labeled peaks in Fig. 5B are the TMT for quantification of this peptide. The reporter ion spectra for two different peptides of CSTB from group I, as shown in Fig. 5C,D, are very consistent, which represented the systematic down regulation of CSTB in cancer patients.

### Gene ontology analysis by PANTHER

**Figure 6:**
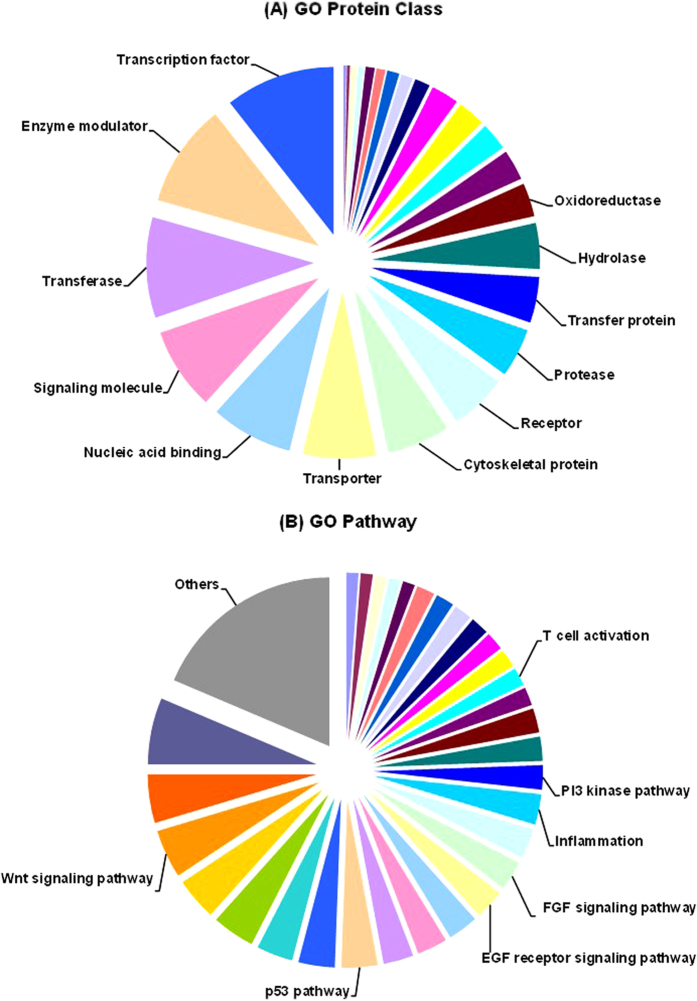
Panther gene ontology pathway analysis of all proteins: (**A**) GO protein class analysis; (**B**) GO pathway analysis.

Protein classification was finished by Panther Classification System based on their molecular function, related biological process, cellular component, protein class and related pathway. The Gene Oncology protein class analysis and pathway analysis of these proteins are shown in Fig. 6A,B, respectively.

### Candidate biomarker verification

**Figure 7:**
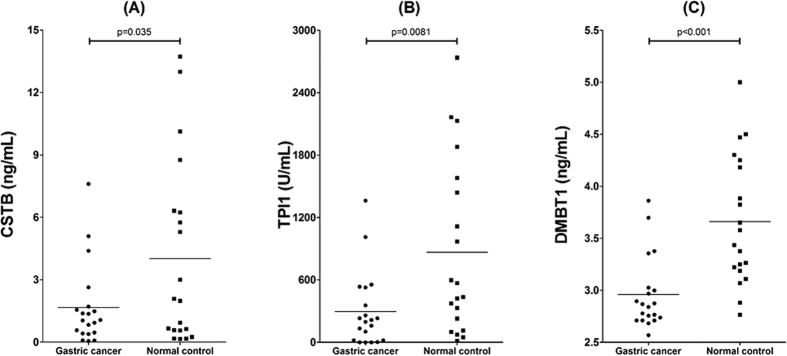
Dot plot for biomarker verification in discovery sample set (n = 40): (**A**) CSTB; (**B**) TPI1; (**C**) DMBT1.

Five proteins were selected for initial biomarker verification, including CSTB, TPI1, DMBT1, CALML3, and ILIRA. ELISA kits were extensively used for the target protein detection and quantification. Three of them were successfully verified in the discovery sample (n = 40) with p < 0.05, including CSTB, TPI1 and DMBT1. The dot plot of these biomarkers in the 20 cancer samples and 20 control samples are shown in Fig. 7.

### Candidate biomarker pre-validation

**Figure 8:**
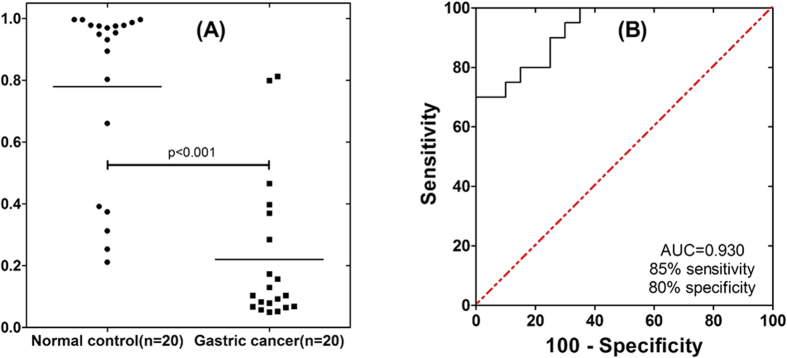
ROC curve and dot plot diagram for the logistic regression model. (**A**) dot plot diagram was based on the ELISA data of the gastric cancer group (n = 20) and healthy control group (n = 20). (**B**) the logistic regression model using 3 biomarkers (CSTB, TPI1 and DMBT1) in the pre-validation sample set results in AUC value of 0.93 with 85% sensitivity and 80% specificity (cutoff, 0.3721).

To further test the utility of these 3 candidates, a new sample set was utilized for pre-validation, which consisted of another 20 cancer samples and 20 control samples. The ELISA results (Supplementary Figure S1) demonstrated that all of them shown significant difference between gastric cancer patients and normal control (p < 0.05). To demonstrate the clinical utility of these salivary proteomic biomarker signatures for gastric cancer detection, logistic regression models were built based on different combinations of biomarkers. Figure 8A is the corresponding dot plot diagram of the three biomarker combination (CSTB, TPI1 and DMBT1) in the 40 pre-validation samples.

### Biomarker performance and utility

Receiver operating characteristic (ROC) curve was built to evaluate the performance of these pre-validated biomarkers, yielding an area under ROC curve (AUC) value between 0.81 and 0.92. The combination of all there biomarkers could yield an AUC value of 0.93 with 85% sensitivity and 80% specificity (Fig. 8B).

## Discussion

### Biomarker discovery for gastric cancer detection

In total, we identified and quantified 519 proteins through the off-line two dimensional LC-MS/MS method (WCX-RPLC). The most abundant protein in saliva was selectively removed by affinity column, which greatly improved the resolution of biomarker discovery. When compared with ref. 37, about 20% of our identified proteins have been discovered by other approaches. Among these quantified proteins, there were 48 proteins shown significant difference between gastric cancer patients and normal subjects. Especially, about one third of these differentially altered proteins are gastric cancer related, either biologically or clinically, which demonstrated that human saliva could be a valuable medium for the detection of gastric cancer.

Of note it that most of these candidate biomarkers were down regulated in the saliva of gastric cancer patients. According to our preliminary work on salivary messenger RNA profiling and salivary microbial analysis from a similar saliva sample set, most identified candidates (which can differentiate gastric cancer patients from normal control subjects with p < 0.05) were also down regulated in cancer patients (data not shown). The consistency among protein, messenger RNA and microbial shown that there are some systematic changes occurred in human body that regulated by remote gastric cancer, which is fulfill the prospective of system biology.

Tumor-secreted exosomes have been found as a key player in determining cancer’s organotropic metastasis^44^. We proposed the role of cancer-derived exosomes in salivary biomarker development for systemic diseases and tested it *in vitro*^45^ and *in vivo*^46^. We found that suppression of exosome biogenesis result in the ablation of discriminatory salivary biomarker development, which might explain why saliva could be used for the detection of distal systemic disease, like gastric cancer.

### The down-regulation of salivary biomarkers in cancer

Through initial verification in the discovery sample set and further confirmed in the pre-validation sample set, three proteins were consistently confirmed by ELISA. CSTB is an inhibitor of cathepsin proteases, which are increased in cancer. The protein levels of CSTB have been shown to correlate with tumor presence and stages. It has also been identified as a potential serum marker in hepatocellular carcinoma^47^. CSTB is a tissue and urinary biomarker for bladder cancer recurrence and disease progression^48^.

Through functional proteomics analysis, TPI1 has been identified in human gastric cancer cells as an anti-drug resistance agent^49^. It was also significantly regulated by *H. Pylori* in human gastric epithelial AGS cells^7^.

DMBT1 is a gene that is located at chromosome 10q 25.3–26.1, a possible tumor suppressor locus indicated by refinement of the losses of heterozygosity in various cancers^50^. The loss of DMBT1 expression may preferentially take place in well-differentiated gastric carcinoma. However, an upregulation of DMBT1 expression is more frequently found across all gastric cancer types^51^.

Human calmodulin-like protein (hCLP), is an epithelial-specific Ca^2+^-binding protein whose expression is strongly down regulated in cancers. Loss of immunoreactivity for human calmodulin-like protein is an early event in breast cancer development. The tumor-sensitive calmodulin-like protein is a specific light chain of human unconventional myosin X^52^. We also found that CALML3 down-regulated significantly in gastric cancer patients.

### Diagnostic utility of salivary biomarkers

The diagnostic utility of these pre-validated biomarkers were evaluated by building the ROC curve and calculate their performance. By combining the three biomarkers through logistic regression, the biomarker panel could reach AUC value of 0.93 with 85% sensitivity and 80% specificity. The results collectively demonstrated that it is very promising to set up a saliva test for the detection of gastric cancer through using these developed biomarkers.

## Conclusion

To the authors’ best knowledge, this is the first *de novo* proteomics biomarker discovery in human saliva for the detection of gastric cancer. New approaches and strategies were engaged for gastric cancer biomarker discovery. Through two phases biomarker development, 48 proteins were successfully discovered through amylase depletion and high throughput quantitative proteomic technology. ELISA further confirmed the presence of three candidates in the cancer saliva. Their performance for the detection of gastric cancer was evaluated, which is very encouraging for further definitive validation. Relay on the point of care technology, salivary diagnostic could be an ideal alternative way for the early detection and screening of gastric cancer.

## Materials and Methods

### Patients and samples

Our biomarker development consisted of two phases, including biomarker discovery phase and biomarker pre-validation phase (Fig. 1A). In total, 40 cancer patients and 40 normal control subjects were recruited for this study. All the saliva samples were collected under a protocol approved by institutional review board (IRB) of Samsung Medical Center and UCLA. All patients provided written informed consents. The methods were carried out in accordance with the approved guidelines. All experimental protocols were approved by Samsung Medical Center and UCLA Medical Centre Ethics Committee. Unstimulated saliva samples were consistently collected, processed, and stabilized as previously described^40^,^41^. All the samples were kept at −80 °C prior to assay. Identified proteomic biomarkers were first verified in the discovery sample set (20 gastric cancer samples and 20 healthy control samples) and then pre-validated in another sample set (20 gastric cancer samples and 20 healthy control samples).

### Sample preparation

Saliva protein concentration was determined by BCA Protein Assay Kit (Thermo Scientific, Rockford, IL, USA). By taking 300 μg of proteins from each individual sample, every four samples were pooled into one sample in cancer group and healthy control group, respectively, thus 5 pool cancer samples and 5 pool healthy control samples were prepared. All the 10 pooled samples were subjected to potato starch affinity column for efficiently removal of alpha amylase as previously described^53^. Briefly, homemade affinity column packed with potato starch (Sigma Aldrich, Saint Louis, USA) was used to trap amylase and the flow through were collected for further analysis. Equal amount of protein from each pool sample was then used for the following experiment. Two pooled saliva samples were made from all the 10 pooled samples as a GIS for the comparison between two TMT-6plex experiments. 1D SDS-PAGE and 2D-DIGE were run as previously described^41^ to test the efficiency of amylase removal.

### Reduction, alkylation, digestion, and labeling with TMT of the saliva samples

For each TMT-6plex experiment, 100 μg proteins in each pool saliva sample were dissolved in 45 μL of 200 mM TEAB. The sample was adjusted to a final volume of 100 uL with ultrapure water. With adding 5 μL 200 mM TCEP, the reaction was performed for 1 hour at 55 °C. Then, 5 μL of 375 mM IAA was added, and the mixture were reacted for 30 min in the dark at room temperature. 5 μL of freshly prepared trypsin (Promega, WI, USA) at 0.5 μg/uL concentration in TEAB (200 mM) was added. The digestion was performed overnight at 37 °C.

In group I, 1 GIS, 3 healthy control samples and two cancer samples were labelled by TMT with reporters at m/z = 126.1, 127.1, 128.1, 129.1, 130.1, 131.1, respectively (Fig. 1B). In group II, 1 GIS, the left 3 cancer samples and 2 healthy controls samples were labelled by another set of TMT with reporters at m/z = 126.1, 127.1, 128.1, 129.1, 130.1, 131.1, respectively. After 1 h of reaction at RT, 8 μL of 5% hydroxylamine (w/v) was added in each tube and mixed for 15 min. The six samples in each group were pooled into a new tube, respectively, and dried for storage at −80 °C.

### Cation exchange fractionation of the pooled TMT-labeled saliva peptides

The pooled TMT-labelled saliva peptides were fractionated by cation-exchange chromatography using a flow rate at 0.8 mL/min on a 4.6 mm × 250 mm (5 μm, 125 Å) TSK gel CM-2SW column (Tosoh Bioscience, Stuttgart, Germany). The gradient was run as follows: 0–3 min 100% A (10 mM ammonium acetate, 25% acetonitrile, adjusted to pH = 3 with HAC), then to 100% B (200 mM ammonium acetate, 25% acetonitrile, adjusted to pH = 3 with HAC) at 15 min. As shown in Supplementary Figure S2, fractions were collected every minute and desalted via PepCleanTM C18 spin columns (Pierce, Rockford, IL, USA). The 12 fractions were dried under vacuum and stored at −80 °C for further LC-MS/MS analysis.

### LC-MS/MS analysis

Peptides in each fraction were rehydrated in 2% (v/v) acetonitrile/0.1% (v/v) formic acid in water and injected with an autosampler (Eksigent NanoLC-2D, CA, USA). Peptides were first enriched on a reverse phase trap column (ProteoPep II, 100 μm × 2.5 cm, C18, 5 μm, 300 Å, New Objective, USA) and then eluted to analytical column (Magic C18AQ, 100 μm × 15 cm, 3 μm, 200 Å, Michrom Bioresources, USA). The mobile phase consisted of buffer (A) 2% acetonitrile and 0.1% formic acid in water, and buffer (B) 2% water and 0.1% formic acid in acetonitrile. A flow rate of 250 nL/min was applied for the separation of peptides for 140 mins. The gradient run was follows: 0–1 min, 2% B, then to 30% B at 90 min, 80% B at 110 min, and 2% B at 140 min. The mass spectrometer voltage was set to 1800 V and the heated capillary was kept at 180 °C. All mass spectra were acquired in the positive ionization mode with m/z scan range of 350–2000. The LTQ-Orbitrap XL (Thermo Fisher Scientific, San Jose, USA) was operated in a top 6 configuration at 60,000 resolving power (defined by m/Δm50%) for a full scan, with enabled charge state screening, monoisotopic precursor selection enabled, and + 1, and unassigned charge states rejected. After master scan, three most intense ions were subjected for collision-induced dissociation (CID) fragmentation using an isolation window of 3.0, collision energy of 30, default charge state of 2 and activation time of 30 ms. Fragmentation of three most intense TMT-reporter-labelled ions was achieved with HCD fragmentation at 7500 resolving power in the LTQ-Orbitrap using an isolation window of 2, collision energy of 40, default charge state of 2 and activation time of 30 ms.

### Protein identification and quantification

LC-MS/MS data analysis was performed with Qual Brower (v2.0.7) and Proteome Discoverer (v1.3) interfaced SEQUEST (Human IPI database v3.78, 302626 entries). Up to two missed cleavage sites were allowed during the database search. Peptides and proteins identification were filtered with charge state dependent cross correlation (Xcorr) ≥2.0 and peptide rank No. 1 with requiring at least two peptides per protein. The filters allowed a 99% confidence level of protein identification with less than 1% false discovery rate. The Reporter Ions Quantitizer in the Proteome Discoverer was used to quantify the TMT reporter ion intensities at 126.13–131.14 *m/z*. Protein identification and quantification intensity ratios were exported to Microsoft Excel software. Reporter ion isotope correction factors were applied by subtracting the contribution of reporter ion isotopes to adjacent reporter ion intensities and adding these intensities back to the proper channel, after which data were normalized by median intensities for subsequent analyses.

### ELISA

The ELISA tests for CSTB, TPI1 and DMBT1 (Antibodies-online, Atlanta, GA, USA) were performed according to the manufacturers’ instructions. All saliva samples were diluted 5 times with sample diluents for all three proteins.

### Data analysis

The Graphpad Prism (Version 5.01) was used for all data analysis. For the number of proteins quantified in the 10 samples, p value was calculated based on t test and p < 0.05 was used as the cut-off for significance. The ROC curve and AUC value were constructed by numerical regression of the ROC curve. The confirmed gastric cancer related proteins were fitted for logistic regression models. Protein classification was finished by Panther Classification System (database version 6.1) based on their molecular function, related biological process, cellular component, protein class, and related pathway.

## Additional Information

**How to cite this article**: Xiao, H. *et al.* Differential Proteomic Analysis of Human Saliva using Tandem Mass Tags Quantification for Gastric Cancer Detection. *Sci. Rep.* **6**, 22165; doi: 10.1038/srep22165 (2016).

## Supplementary Material

Supplementary Information

## Figures and Tables

**Table 1:** Patient Demographics and Clinical Profiles.

Demographic	Discovery Set		Confirmation Set	
	Cancer (n = 20)	Control (n = 20)	Cancer (n = 20)	Control (n = 20)
Age, years	54.95 ± 10.82	56.10 ± 9.80	54.45 ± 11.14	44.45 ± 12.54
Range	33–76	38–72	30–76	26–67
Sex				
Male	8	8	12	12
Female	12	12	8	8
Ethnicity				
Korean	20	20	20	20
Smoke History				
Yes	3	3	10	6
Drinking				
Yes	6	5	8	9
H. pylori infection				
Yes	14	5	12	3
Intestinal metaplasia				
Yes	6	5	7	0
T stage				
I, II, III, IV	12, 3, 4, 1		13, 3, 2, 2	

**Table 2:** Top ranked salivary protein biomarker candidates.

Accession	Description	# Peptides	MW [kDa]	pI	Ratio (cancer/control)	p value (cancer vs. control)
IPI00216984.5	Calmodulin-like protein 3	6	16.9	4.42	0.575	0.008
IPI00021828.1	Cystatin-B	345	11.1	7.56	0.579	0.025
IPI00409567.2	Polycystin-1	3	461.1	6.77	0.584	0.015
IPI00419920.2	carboxylesterase 2	4	67.0	6.57	0.606	0.021
IPI00174541.1	Interleukin-1 receptor antagonist protein	7	16.1	4.88	0.616	0.014
IPI00013384.4	Kinase suppressor of Ras 1	2	96.9	8.57	0.621	0.026
IPI00788666.3	Lipoxygenase homology domain-containing protein 1	4	252.4	5.43	0.633	0.000
IPI00797270.4	Triosephosphate isomerase	39	26.7	6.90	0.640	0.030
IPI00456853.4	WASH complex subunit FAM21C	2	143.6	4.79	0.640	0.031
IPI00619925.2	Centromere-associated protein E	7	301.6	5.57	0.643	0.027
IPI00479345.2	Cancer-associated gene 1 protein	6	74.6	5.99	0.645	0.035
IPI00012505.7	Transmembrane protease serine 13	3	57.1	8.60	0.646	0.025
IPI00018294.3	Zinc finger protein Rlf	46	217.8	6.77	0.651	0.009
IPI00070943.4	Phosphatidylinositol 4-kinase alpha	2	231.2	6.87	0.656	0.000
IPI00001159.11	Translational activator GCN1	2	292.6	7.47	0.656	0.016
IPI00604778.1	Vacuolar protein sorting-associated protein 13C	2	402.8	6.38	0.658	0.045
IPI00218684.3	Short of Autism susceptibility gene 2 protein	15	136.3	9.35	0.659	0.021
IPI00290566.1	T-complex protein 1 subunit alpha	16	60.3	6.11	0.671	0.027
IPI00872278.1	Deleted in malignant brain tumors 1 protein	57	273.3	5.44	0.673	0.004
IPI00413365.3	Zinc finger protein 318	4	251.0	7.20	0.675	0.004
IPI00025753.2	Desmoglein-1	9	113.7	5.03	0.687	0.041
IPI00478657.4	G-rich sequence factor 1	2	53.1	6.19	0.690	0.009
IPI00798373.3	Bardet-Biedl syndrome 4 protein	2	58.2	7.31	0.698	0.024
IPI00298994.6	Talin-1	2	269.6	6.07	0.699	0.003
IPI00972974.1	53 kDa protein	5	52.8	5.48	0.703	0.032
IPI00903081.1	Ras-associating and dilute domain-containing protein	2	117.4	7.09	0.709	0.032
IPI00744872.3	Protein FAM179B	2	189.2	8.50	0.715	0.019
IPI00894030.2	258 kDa protein	4	257.8	8.54	0.716	0.041
IPI00555812.5	Vitamin D-binding protein	2	52.9	5.45	0.718	0.031
IPI00384972.3	MLL1/MLL complex subunit KIAA1267	2	120.9	8.75	0.719	0.002
IPI00795394.3	Dynein heavy chain 9, axonemal	2	503.0	5.91	0.719	0.025
IPI00844048.1	Protein C1orf14	2	60.2	5.31	0.750	0.005
IPI00219168.7	Spectrin beta chain, brain 4	7	416.6	6.67	0.759	0.038
IPI00940245.1	Immunoglobulin heavy chain variant	673	44.8	6.13	0.778	0.038
IPI00386879.1	cDNA FLJ14473 fis	673	53.1	6.86	0.778	0.038
IPI00008173.2	Pleckstrin homology domain-containing family G member 1	2	155.3	6.24	0.788	0.021
IPI00151988.4	Zinc finger protein 532	2	141.6	8.65	0.795	0.026
IPI00647704.1	cDNA FLJ41552 fis	652	53.3	6.52	0.802	0.044
IPI00947235.1	8 kDa protein	67	8.2	8.56	0.836	0.005
IPI00874215.2	cDNA FLJ59298	2	134.1	5.44	0.841	0.015
IPI00759613.3	Titin isoform N2-A	36	3711.3	6.52	0.857	0.038
IPI00735451.4	Immunoglobulin heavy chain	29	12.7	8.27	1.131	0.011
IPI00923519.1	Protein unc-80 homolog	2	337.6	7.06	1.207	0.039
IPI00026089.4	Splicing factor 3B	2	145.7	7.09	1.561	0.021
IPI00219076.1	Signal transducer and activator of transcription 2	3	74.6	7.15	1.733	0.026
IPI00947307.1	cDNA FLJ58075	2	108.8	5.77	1.835	0.006
IPI00909737.1	cDNA FLJ55140	2	55.5	4.84	1.992	0.008
IPI00012024.1	Histatin-1	9	7.0	9.13	3.984	0.044
