# Supplementary material for: Differential Proteomic Analysis of Human Saliva using Tandem Mass Tags Quantification for Gastric Cancer Detection
Source: Sci Rep. 2016 Feb 25;6:22165. doi: 10.1038/srep22165 (PMC4766442; doi:10.1038/srep22165)
Supplement: Supplementary Information [file srep22165-s1.doc]

Supplementary Information for

**Differential Proteomic Analysis of Human Saliva using Tandem Mass Tags Quantification for Gastric Cancer Detection**

Hua Xiao ^a^,*, Yan Zhang ^b^, Yong Kim ^c^, Sung Kim ^d^, Jae Joon Kim ^e^, Kyoung Mee Kim ^f^, Janice Yoshizawa ^c^, Liu-Yin Fan ^a^, Cheng-Xi Cao ^a^, and David T.W. Wong ^c^, *

**Figure S1.** Dot plot for biomarker pre-validation in the pre-validation sample set (n=40, including 20 cancer samples and 20 control samples): (A) CSTB; (B) TPI1; (C) DMBT1.

**Figure S2.** Chromatogram for the separation of salivary peptides through WCX. Absorbance at 280 nm was collected after wavelength optimization. Fractions were collected every minute.
